# Matrix Metalloproteinases and Minocycline: Therapeutic Avenues for Fragile X Syndrome

**DOI:** 10.1155/2012/124548

**Published:** 2012-05-20

**Authors:** Saul S. Siller, Kendal Broadie

**Affiliations:** ^1^Departments of Biological Sciences and Cell and Developmental Biology, Kennedy Center for Research on Human Development, Vanderbilt University Station B, Nashville, TN 37232, USA; ^2^Stony Brook School of Medicine, Stony Brook University, Stony Brook, NY 11794, USA

## Abstract

Fragile X syndrome (FXS) is the most common known genetic form of intellectual disability and autism spectrum disorders. FXS patients suffer a broad range of other neurological symptoms, including hyperactivity, disrupted circadian activity cycles, obsessive-compulsive behavior, and childhood seizures. The high incidence and devastating effects of this disease state make finding effective pharmacological treatments imperative. Recently, reports in both mouse and *Drosophila* FXS disease models have indicated that the tetracycline derivative minocycline may hold great therapeutic promise for FXS patients. Both models strongly suggest that minocycline acts on the FXS disease state via inhibition of matrix metalloproteinases (MMPs), a class of zinc-dependent extracellular proteases important in tissue remodeling and cell-cell signaling. Recent FXS clinical trials indicate that minocycline may be effective in treating human patients. In this paper, we summarize the recent studies in *Drosophila* and mouse FXS disease models and human FXS patients, which indicate that minocycline may be an effective FXS therapeutic treatment, and discuss the data forming the basis for the proposed minocycline mechanism of action as an MMP inhibitor.

## 1. Introduction

Fragile X syndrome (FXS), the most common heritable cause of intellectual disability and autism spectrum disorders [1], has a prevalence of roughly 1 in 4000 males and 1 in 6000 females [2, 3]. While a hallmark of FXS is low IQ (~40), patients present with a wide spectrum of behavioral, physical, and neurological symptoms [4–6]. Behavioral problems include hyperactivity and hypersensitivity to sensory stimuli, anxiety and mood disorders, disrupted sleep patterns, defects in cognitive learning and memory consolidation, and impaired social skills [1, 4, 5, 7–14]. Often, a speech deficit is the first symptom leading to a FXS clinical diagnosis [4, 5]. Although there are usually not significant nonneurological medical impairments associated with the syndrome, FXS patients typically display male macroorchidism, macrocephaly with prominent ears and a long, thin face, joint hypermobility, and flat feet [4, 5, 15–17]. Elevated electroencephalogram (EEG) activity is characteristic, and epileptic seizures are present in ~20% of FXS patients, typically with remittance by adulthood [18].

FXS is caused by loss of the *fragile X mental retardation 1* (*FMR1*) gene product, FMRP [19], typically due to expansion (>200) of the CGG trinucleotide repeat in the 5′-untranslated gene region, leading to subsequent hypermethylation and gene silencing [20]. FMRP is an mRNA-binding protein known to regulate mRNA stability, trafficking, and translation [21–26], with roles in the activity-dependent regulation of synaptic development and plasticity [26–33]. Numerous studies support the “mGluR theory of FXS” that suggests enhanced group 1 metabotropic glutamate receptor 5 (mGluR5) signaling is responsible for deficits in synaptogenesis, dendritic spine morphology, long-term potentiation (LTP) and depression (LTD) in the disease state [5, 27, 28, 30–32, 34–43]. Consequently, many studies have focused on mGluR inhibitors, such as 2-methyl-6-phenylethynyl-pyridine (MPEP), as a therapeutic intervention for FXS [31, 38–40, 43–47], with considerable success. For example, in the *Drosophila* disease model, MPEP effectively prevents cellular synaptic deficits and behavioral learning and memory impairments [31, 42, 45, 46]. While some mGluR inhibitors cannot be used in FXS patient treatment due to toxicity and bioavailability limitations (e.g., MPEP), other drugs, such as the selective mGluR5 inhibitor fenobam, are currently in human clinical trials [48, 49]. Lithium, an inhibitor of GSK3*β*, a downstream effector of mGluR5 signaling, is also being taken to clinical trials with promising results as a potential FXS therapeutic treatment [50].

In addition to the promise of mGluR5 pathway interventions, several recent reports suggest minocycline as another potential avenue of FXS therapeutic treatment [51–54]. Used for decades as an antibiotic and acne treatment, the second-generation, semisynthetic tetracycline derivative minocycline has a long half-life, highly lipophilic characteristics, and easily crosses the blood-brain barrier [55]. In addition to its antibiotic actions, minocycline also functions as an anti-inflammatory agent via inhibition of several molecules, including COX-2, iNOS, and p38 MAPK, and as an antiapoptotic agent via inhibition of caspases, among many other putative modes of action [55, 56]. Central to its role in FXS, minocycline is known to inhibit matrix metalloproteinases (MMPs), a family of secreted and membrane-tethered zinc-dependent extracellular proteases with roles in tissue remodeling and intercellular signaling [57–60]. Functioning through one or more of these modes of action, minocycline has been shown to have neuroprotective effects [55] and has been previously suggested to be useful in the treatment of several neurodegenerative diseases, including multiple sclerosis [61], amyotrophic lateral sclerosis (ALS) [62, 63], Huntington's disease [64], Parkinson's disease [65], and Alzheimer's disease [66].

In the FXS disease state, several recent studies have proposed that minocycline exerts its therapeutic effects via MMP inhibition [51, 53]. In humans, a diverse array of at least 24 MMPs functions to cleave components of the extracellular matrix (ECM), including both secreted and cell membrane proteins [57–59]. In general, MMPs contain a pro domain that is cleaved to activate the extracellular protease, an enzymatic zinc-containing catalytic domain, a linker domain, and a hemopexin domain [57–59]. MMPs are part of the metzincin family of proteases, named for a conserved methionine residue and zinc in the protease catalytic sites, which includes ADAMs (a disintegrin and metalloproteinase) and ADAM proteases with thrombospondin motifs (ADAMTSs) [57–59, 67]. In the central nervous system, MMPs have been implicated in axonal guidance, synaptogenesis, neurotransmission, synaptic plasticity, and behavioral learning [57, 58, 67]. MMPs are endogenously inhibited by tissue inhibitors of MMPs (TIMPs), with 4 family members in humans [68, 69]. Along with MMPs, TIMPs are also expressed in the central nervous system, where they also regulate synaptic mechanisms and behavioral outputs [67–69].

In this paper, we review recent evidence that minocycline acts as an effective therapeutic treatment in FXS genetic animal models and human FXS patient clinical trials. We then summarize current data supporting an “MMP inhibition mechanism” by which minocycline may remediate the FXS disease state. Finally, we consider possible pathways through which the MMP/TIMP and FMRP pathways could intersect in the formation of FXS pathogenesis.

## 2. Minocycline in Fragile X Animal Models

 Minocycline was first revealed as a possible FXS therapeutic treatment in the mouse disease model [51]. The *FMR1* knockout (KO) mouse is a well-validated model of the human disease state [30, 40, 51, 71, 72], which displays macroorchidism, hyperactivity, and some learning/memory deficits [71–73]. As with cortical postsynaptic dendritic spine morphogenesis defects in FXS patient brain autopsies [74, 75], *FMR1* KO mice exhibit an immature dendritic spine profile with more long, thin filopodia-like spines and a proportional lack of mature short, stubby, mushroom-like spines [76–79]. This synaptic maturation/overgrowth defect has been reported to vary in severity/penetrance in different brain regions and may be developmentally transient, with the defect being most prominent during neural circuit refinement stages of early postnatal development [80, 81]. The dendritic spine phenotype of *FMR1 *KO mice has been linked with defects in postsynaptic group 1 mGluR signaling activity, as with defects in synaptic plasticity [82]. This postsynaptic dendritic spine defect has long been considered the FXS neuroanatomical hallmark and was the logical choice to first assay the effects of minocycline treatment.

 In 2009, Bilousova and colleagues provided the first evidence for the therapeutic effectiveness of minocycline in the *FMR1* KO mouse with a thorough examination of dendritic spine profiles of hippocampal neurons both *in vitro *and *in vivo *[51]. This study showed that 20 *μ*M minocycline promoted maturation of dendritic spines in control hippocampal neuronal cultures. In *FMR1* KO neurons, minocycline treatment in culture (20 *μ*M for 17 hours) or fed to mice (30 mg/kg/day in drinking water) shifted the immature dendritic spine profiles towards normal dendritic spine profiles (Table 1). It is important to note that no change was reported in dendritic spine length or the total number of spines between *FMR1* KO untreated and treated conditions; rather, there was solely a proportional shift of the number of immature to mature spines upon minocycline treatment [51]. Following this neuroanatomical analysis, the same study examined the effect of minocycline treatment on *FMR1* KO mouse behavior [51]. Minocycline was again fed to newborn mice via their drinking water at levels of 30 mg/kg/day. At 3 weeks of age, treated *FMR1* KO mice were found to be less anxious in an elevated plus maze assay (Table 1). Moreover, minocycline treatment resulted in better memory in a passive Y maze, compared to untreated *FMR1 *KO mice [51]. Additionally, a follow-up study was very recently conducted by this same group [70]. In *FMR1* KO adult male mice, it was found that the rate of ultrasonic vocalizations (USVs) produced during mating is reduced. No other acoustic property deficits were identified. Interestingly, minocycline treatment restored a normal rate of USVs in the *FMR1* KO mouse (Table 1). Thus, minocycline was shown to be effective at ameliorating both neuroanatomical and behavioral FXS defects in the mouse disease model.

Recently, a study employing the *Drosophila* FXS disease model corroborated and expanded these data, reinforcing the therapeutic potential of minocycline treatment [53]. Over more than a decade of research, the *Drosophila* system has been firmly established as a highly validated model of the human disease state [25, 26, 31, 42–47, 83–99]. *Drosophila* contains a single *FMR1 *homolog, *dFMR1*, compared to the three Fragile X family genes present in mammals (*FMR1*, *FXR1,* and *FXR2*). Only human *FMR1* rescues the broad range of neurological phenotypes caused by *dFMR1* KO, with human *FXR1/2* having no activity, showing that *FMR1* function has been evolutionarily conserved and that human *FMR1* requirements can be effectively dissected in the *Drosophila* FXS disease model [84, 99]. Like human patients and the mouse model, *dFMR1* KO flies exhibit synaptic overgrowth and synaptic immaturity in a range of neural circuits, including motor neurons [84, 86, 99], clock neurons [84, 87, 88, 99], and learning/memory neurons [26, 42]. Likewise mimicking the human disease condition, *dFMR1* null animals display macroorchidism and deficits in spermatogenesis, hyperactivity and circadian arrhythmicity, and strong deficits in learning formation and memory consolidation [46, 83, 85, 87, 88, 92]. The breadth and FXS disease relevance of these phenotypes makes *Drosophila* an excellent system to examine minocycline's effectiveness in treating the disease state.

In 2011, Siller and Broadie provided a follow-up study examining the therapeutic effectiveness of minocycline in the *dFMR1* KO in a thorough examination of synaptic architecture in a range of disparate neural circuit types [53]. Synapse structure was analyzed in three locations: (1) the well-characterized glutamatergic neuromuscular junction (NMJ) in the peripheral musculature, (2) the pigment dispersing factor (PDF) neuropeptidergic small ventrolateral (sLN_v_) clock neurons in the central brain, and (3) Kenyon cell neurons of the brain mushroom body (MB) learning and memory center (Table 1). In all three circuit types, the *dFMR1* KO displays the same characteristic synaptic overgrowth and overelaboration, including an expanded synaptic arbor domain, increased synaptic branching, and increased supernumerary synaptic boutons with prominent structurally immature synaptic sites [25, 26, 42, 47, 86, 87]. Minocycline was fed to *dFMR1* KO mutants in their food during larval development in the range of 2–20 *μ*M and to adults at the higher dosage of 1 mM (Table 1), resulting in a dosage-dependent improvement of synaptic architecture towards the wildtype state [53]. Interestingly, the CNS synaptic deficits responded better to minocycline treatment than the NMJ defects, although the reason for this difference is currently unknown. At the NMJ, minocycline treatment completely prevented the accumulation of immature synaptic boutons and partially prevented the overabundance of mature boutons, but had no effect on the defect in synaptic branching in *dFMR1* KOs [53]. In the brain, both in clock neurons and in MB Kenyon cells, minocycline treatment both prevented the excess synaptic branching and completely rescued the overelaboration of synaptic boutons in *dFMR1* KOs (Table 1). Unlike the mouse study, the *Drosophila* study found no effect of minocycline treatment on wildtype synaptic architecture [53]. Moreover, the *Drosophila* study contained no behavioral analyses. However, at a neuroanatomical level, the *Drosophila* study corroborates the findings of the mouse study and adds further evidence for the effectiveness of minocycline as a broad-spectrum FXS therapeutic treatment in multiple classes of neural circuits [51, 53].

## 3. Minocycline in Fragile X Patients

 Taken together, the two animal model studies strongly suggest minocycline may be an effective FXS treatment. However, the obvious question still remains: does the drug effect translate to human FXS patients? In 2010, two studies began to provide insight into this question with early clinical trials [52, 54]. Utari and colleagues studied 50 FXS patients, given minocycline for 2 weeks or longer, mainly to assess the safety of the drug as a FXS treatment [54]. Of the 50 patients examined, 21 reported side effects, with the most common being gastrointestinal problems, including diarrhea and loss of appetite (Table 1). Most patients reported side effects as mild. One patient did experience coloring of the nails, but none reported tooth discoloration. Of the FXS patients followed throughout the course of the study, most displayed improvements in several areas, including language, attention, social communication, and anxiety [54]. A small subset of patients exhibited worsening in two areas: hyperactivity and moodiness (Table 1). Thus, this study suggests minocycline may be effective in FXS treatment with only mild side effects, and the results warrant a followup, controlled study to more closely examine minocycline effectiveness.

 Paribello and colleagues performed an open-label, add-on minocycline treatment trial with 19 FXS patients aged 13–32 years followed for an 8-week treatment period [52]. One patient dropped out due to side effects, and two other patients developed asymptomatic seroconversion of antinuclear antibodies, a diagnostic in autoimmune disorders. None of the other patients reported serious side effects that were attributed to the minocycline treatment, although dizziness, headaches, sleepiness, and diarrhea were reported as mild side effects (Table 1). In this trial, significant improvements occurred in behavioral outcomes using the Aberrant Behavior Checklist-Community Edition (ABC-C) [52]. Four out of five subscale scores showed significant improvement, including the irritability subscale, which was used as the primary outcome measure, and stereotypy, hyperactivity, and inappropriate speech subscales, which were employed as secondary outcome measures (Table 1). Positive results using the clinical global improvement scale (CGI) and the visual analog scale for behavior (VAS) as measures were also reported for the majority of minocycline-treated FXS patients [52]. Interestingly, given the choice to extend minocycline treatment for 1 year, 18 of the 19 families independently decided to continue treatment based on their perceptions of behavioral improvements. Taken together with the Utari study, both sets of data strongly suggest that minocycline is a relatively safe and potentially effective treatment for FXS patients [52, 54]. However, a double-blind, placebo-controlled clinical treatment trial is still necessary to provide concrete evidence that minocycline is a positive and effective FXS therapy.

## 4. Mechanism of Action: MMP Inhibition

**Figure 1:**
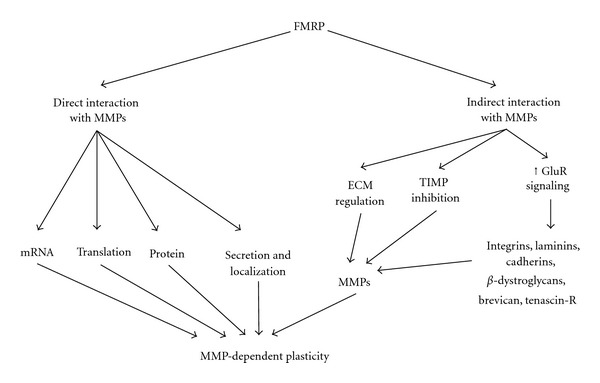
Schematic of potential interaction mechanisms between FMRP and MMPs. FMRP may more directly regulate MMPs at the level of transcript stability, translation, or protein function. FMRP may more indirectly convergently interact with MMPs in the regulation of extracellular matrix (ECM) components and their receptors, via the endogenous TIMP regulatory mechanism, or via glutamate receptor (GluR) signaling.

 The 2009 mouse FXS model study provides good evidence that minocycline acts through inhibition of secreted matrix metalloproteinase-9 (MMP-9) [51]. In other neurological disorders, such as multiple sclerosis, minocycline has similarly been found to be effective as an MMP-9 inhibitor [55]. Bilousova and colleagues performed both Western Blot and gel zymography analyses to assess differences in MMP-9 levels and enzymatic (gelatinase) activity in the mouse hippocampus [51]. In *FMR1 *KO (P7) mice, levels of active MMP-9 as well as MMP-9 gelatinase activity were both increased compared to controls (Figure 1). Importantly, minocycline treatment of the *FMR1* KO decreased both hippocampal active MMP-9 protein levels and hippocampal gelatinase activity towards the wildtype condition [51]. Interestingly, MMP-9 treatment of wildtype hippocampal cell cultures induced immature dendritic spine profiles with a greater proportion of long, thin filopodia-like dendritic spines, mimicking the *FMR1* KO state [51], a finding that has since been validated via genetic methods [100]. Together, this evidence suggests that upregulation of secreted active MMP-9 is a novel aspect of the molecular pathology of FXS and that MMP-9 inhibition is the mechanism of action of minocycline in alleviating FXS phenotypes (Figure 1).

 In the follow-up *Drosophila* study, Siller and Broadie greatly extended testing of this MMP hypothesis, taking advantage of the fly's relative genetic simplicity (2 MMPs in *Drosophila* compared to 24 in mammals; 1 TIMP in *Drosophila* compared to 4 in mammals) [53, 57, 58, 60, 101]. The two *Drosophila* MMPs include secreted MMP-1 and membrane anchored MMP-2, with a good antibody probe available for MMP-1 only. MMP-1 expression levels and gelatinase activity showed no significant differences in the *dFMR1* KO compared to control, at least at the NMJ synapse with immunocytochemistry and *in situ* zymography and in whole-brain Western Blots (Figure 1) [53]. Nevertheless, to begin to test the MMP hypothesis, the endogenous TIMP inhibitor was genetically overexpressed in the *dFMR1* KO background to mimic the proposed minocycline effect. At the NMJ, TIMP overexpression was highly efficacious in suppressing the synaptic structural overelaboration characterizing the *dFMR1* KO, restoring the synaptic branching and excess mature and immature bouton formation to the wildtype condition [53]. Conversely, TIMP overexpression causes early developmental lethality and tracheal deformations prior to death [60, 102, 103], and *dFMR1* removal bidirectionally suppressed these TIMP overexpression phenotypes. Importantly, a *dFMR1*; *MMP-1* double KO mutant displayed the same reciprocal suppression of phenotypes, with prevention of *dFMR1* KO synaptic architecture defects and rescue of *MMP-1* KO tracheal defects and early lethality [53]. Together, these data provide excellent evidence for a specific genetic interaction between the TIMP/MMP-1 and FMRP pathways (Figure 1). Taken with the previous mouse FXS model study showing specific upregulation of secreted MMP-9, reduced upon minocycline treatment, the combined data set strongly suggests that minocycline is inhibiting MMPs to exert its alleviatory actions on FXS phenotypes [51, 53].

A critical question is to determine how the FMRP and TIMP/MMP pathways intersect (Figure 1). FMRP is best known as a negative translational regulator, and, therefore, it is possible that FMRP directly inhibits MMP expression, resulting in MMP upregulation in the FXS disease state. Based on known FMRP functions, this interaction could happen at the level of regulating MMP mRNA stability or translation, or FMRP could secondarily influence MMP protein function, secretion, or localization via acting on MMP-interacting proteins (Figure 1). However, numerous more indirect interactions between the two pathways are also possible. FXS is a disease of enhanced glutamate receptor signaling, associated with defects in synaptic morphogenesis and plasticity (LTD/LTP) [35, 37]. Several studies have shown that NMDA glutamate receptor signaling causes local MMP-9 release, leading to MMP-mediated synaptic plasticity events [104–106], presumably via extracellular proteins, such as integrins, laminins, cadherins, *β*-dystroglycan, brevican, and tenascin-R, which have all been implicated in hippocampal LTP [57, 58, 67] (Figure 1). Indeed, recent work implicates integrin *β*1 signaling as the mechanism by which MMP-9-mediated changes in dendritic spine morphology occur [100]. Furthermore, MMP-dependent synapse remodeling can be blocked by NMDA receptor inhibitors, and NMDA receptor activity has been shown to increase MMP-9 activity, suggesting another possible link [58]. Moreover, MMPs play roles in axonal-dendritic structural remodeling [58, 67]; for example, with MMP-9 present in mammalian dendritic spines and MMP-1 present at the *Drosophila* glutamatergic NMJ [53, 107, 108]. *MMP-9* KO mice display deficiencies in hippocampal LTP, and other changes, for example, after spinal cord injury, can induce elevated gelatinase activity, showing that careful control of MMP expression levels is critical to synaptic regulation [105, 109]. In addition, MMP inhibition may lead to TIMP signaling changes due to decreased levels of MMP-bound TIMP versus increased levels of free unbound TIMP [110]. For example, the balance between MMP-7 and TIMP-1 was recently shown to be important for pro-nerve growth factor (NGF) cleavage and neuroprotection following kainite-induced seizures [111], which could possibly provide a link with seizure manifestations in FXS patients. Thus, while it is unclear how the MMP and FMRP pathways intersect, it is clear that a number of intriguing possibilities need to be explored (Figure 1).

## 5. Conclusions and Future Directions

 FXS is a devastating neurological disease characterized by a broad spectrum of cellular and behavioral symptoms [4, 5]. While much attention has been focused on mGluR5 inhibitors as a potential avenue of disease treatment [31, 45–47], a significant amount of new evidence from *Drosophila* [53], mouse [51], and human studies [52, 54] suggests that the common tetracycline derivative minocycline may be a new and highly effective treatment alternative (Table 1). These recent reports indicate that minocycline may be a broad-spectrum treatment with only mild side effects. The clear next step is to pursue a double-blind, placebo-controlled FXS clinical trial of minocycline effectiveness. Importantly, it may be critical to test minocycline in young children as it is probable that treatment effectiveness may be linked, in part, to developmentally transient events of neural circuit formation and/or refinement. Of course, it is to be hoped that the inherent plasticity of the nervous system will also make adult minocycline treatments effective. Thus, minocycline holds the real possibility of being an accessible and cost-effective broad treatment for the disease. Importantly, minocycline has long been FDA approved, greatly facilitating its rapid dissemination to the FXS community. This is indeed an exciting development for families afflicted by this devastating neurological disease.

Both *Drosophila* and mouse studies point toward MMP inhibition being the mechanism of minocycline action in FXS [51, 53]. However, another possibility that must be considered arises from the fact that minocycline also functions as an antibiotic by inhibiting bacterial translation, and a similar function could be predicted to antagonize the effect of losing FMRP translational repression, causing elevated translation [21]. Although this should not be a consideration at the levels of minocycline used in the recent FXS studies (Table 1), which are the same dosages as used for treatment of acne and bacterial infections, such as neurosyphilis [112, 113], it remains a strong possibility to be investigated as potentially FXS patients may be more sensitive toward minocycline treatment than healthy individuals. Significant research into minocycline's effects on eukaryotic ribosomes, especially in FXS, has not been done and must be performed extensively to determine if the minocycline mechanism of action is through its ability to regulate translation. In addition, the available evidence does not rule out the possibility of other mechanisms of minocycline action, such as p53 MAPK regulation [55, 114, 115]. Nevertheless, the recent *Drosophila* study strongly indicates specific genetic interplay between the MMP and FMRP pathways that should be the focus of investigation in the immediate future [53]. It will be important to fully study the roles of TIMP and MMPs in the context of synaptic development/refinement and to define overlapping and distinct synaptic functions of the different MMP family members (i.e., membrane-anchored versus secreted). Moreover, a great limitation in the recent *Drosophila *study was that it only examined synaptic structure defects, and it is imperative to extend the work to the examination of minocycline/MMP involvement at the level of synaptic function/plasticity and behavioral outputs in the *Drosophila* FXS model. Besides the neuronal mechanisms, it is also important to note that the genetic interaction between MMP and FMRP occurs also in nonneuronal tissues, because *MMP-1* nonneuronal phenotypes and overall lethality are rescued by *dFMR1* removal [53]. A similar nonneuronal *FMR1 *function is also revealed by the joint symptoms of FXS patients, strongly implicating an ECM component of the human disease state. Moving forward, it will be important to understand how TIMP/MMP dysfunction fits into the larger picture of FXS pathogenesis, to provide both a greater understanding of TIMP/MMP roles at the synapse, as well as to bring to light possible new therapeutic targets for FXS.

## Figures and Tables

**Table 1:** Summary of recent minocycline treatment trials in *Drosophila* and mouse disease models, and human clinical studies. The columns display the systems, minocycline dosages, phenotypes tested, treatment outcomes and side effects.

Model	Dosage	Phenotypes tested	Treatment effects	Side effects	Study
*Drosophila*	20 *μ*M in larvae; 1 mM in adult (oral feeding)	Synpatic structure of NMJ, sLN_v_, and MB neurons	Prevention of all neuroanatomical defects	None	Siller and Broadie, 2011 [53]
Mouse	20 *μ*M for 17 hrs *in vitro*; 30 mg/kg/day *in vivo *	Immature dendritic spine profiles, anxiety, memory defects, decreased rate of USVs	More mature dendritic spine profiles, less anxious, memory improvements, increased rate of USVs	More mature dendritic spines	Bilosuova et al., 2009; [51] Rotschafer et al., 2012 [70]
Human	50 mg BID (low dose) 100 mg BID (high dose)	Behavioral symptoms	Better language and social communication skills, less anxiety, more attentive; Better irritability, stereotypy, hyperactivity, inappropriate speech subscales on ABC-C	GI issues, diarrhea, loss of appetite, dizziness, headaches	Utari et al., 2010; [54] Paribello et al., 2010 [52]

## References

[1] Koukoui SD, Chaudhuri A (2007). Neuroanatomical, molecular genetic, and behavioral correlates of fragile X syndrome. *Brain Research Reviews*.

[2] Crawford DC, Acuna JM, Sherman SL (2001). FMR1 and the fragile X syndrome: human genome epidemiology review. *Genetics in Medicine*.

[3] Hagerman PJ (2008). The fragile X prevalence paradox. *Journal of Medical Genetics*.

[4] Garber KB, Visootsak J, Warren ST (2008). fragile X syndrome. *European Journal of Human Genetics*.

[5] Penagarikano O, Mulle JG, Warren ST (2007). The pathophysiology of fragile X syndrome. *Annual Review of Genomics and Human Genetics*.

[6] Merenstein SA, Sobesky WE, Taylor AK, Riddle JE, Tran HX, Hagerman RJ (1996). Molecular-clinical correlations in males with an expanded FMR1 mutation. *American Journal of Medical Genetics*.

[7] Brown WT, Jenkins EC, Cohen IL (1986). fragile X and autism: a multicenter survey. *American Journal of Medical Genetics*.

[8] Cornish K, Sudhalter V, Turk J (2004). Attention and language in fragile X. *Mental Retardation and Developmental Disabilities Research Reviews*.

[9] Einfeld S, Hall W, Levy F (1991). Hyperactivity and the fragile X syndrome. *Journal of Abnormal Child Psychology*.

[10] Elia M, Ferri R, Musumeci SA (2000). Sleep in subjects with autistic disorder: a neurophysiological and psychological study. *Brain and Development*.

[11] Hagerman RJ, Jackson AW, Levitas A (1986). An analysis of autism in fifty males with the fragile X syndrome. *American Journal of Medical Genetics*.

[12] Kaufmann WE, Cortell R, Kau ASM (2004). Autism spectrum disorder in fragile X syndrome: communication, social interaction, and specific behaviors. *American Journal of Medical Genetics A*.

[13] Miano S, Bruni O, Elia M (2008). Sleep phenotypes of intellectual disability: a polysomnographic evaluation in subjects with Down syndrome and Fragile-X syndrome. *Clinical Neurophysiology*.

[14] Tsiouris JA, Brown WT (2004). Neuropsychiatric symptoms of fragile X syndrome: pathophysiology and pharmacotherapy. *CNS Drugs*.

[15] Chudley AE, Hagerman RJ (1987). fragile X syndrome. *Journal of Pediatrics*.

[16] Hagerman RJ, Van Housen K, Smith ACM, McGavran L (1984). Consideration of connective tissue dysfunction in the fragile X syndrome. *American Journal of Medical Genetics*.

[17] Lachiewicz AM, Dawson DV (1994). Do young boys with fragile X syndrome have macroorchidism?. *Pediatrics I*.

[18] Musumeci SA, Hagerman RJ, Ferri R (1999). Epilepsy and EEG findings in males with fragile X syndrome. *Epilepsia*.

[19] Pieretti M, Zhang F, Fu YH (1991). Absence of expression of the FMR-1 gene in fragile X syndrome. *Cell*.

[20] Sutcliffe JS, Nelson DL, Zhang F (1992). DNA methylation represses FMR-1 transcription in fragile X syndrome. *Human Molecular Genetics*.

[21] Laggerbauer B, Ostareck D, Keidel EM, Ostareck-Lederer A, Fischer U (2001). Evidence that fragile X mental retardation protein is a negative regulator of translation. *Human Molecular Genetics*.

[22] Li Z, Zhang Y, Ku L, Wilkinson KD, Warren ST, Feng Y (2001). The fragile X mental retardation protein inhibits translation via interacting with mRNA. *Nucleic Acids Research*.

[23] Lu R, Wang H, Liang Z (2004). The fragile X protein controls microtubule-associated protein 1B translation and microtubule stability in brain neuron development. *Proceedings of the National Academy of Sciences of the United States of America*.

[24] Muddashetty RS, Kelić S, Gross C, Xu M, Bassell GJ (2007). Dysregulated metabotropic glutamate receptor-dependent translation of AMPA receptor and postsynaptic density-95 mRNAs at synapses in a mouse model of fragile X syndrome. *Journal of Neuroscience*.

[25] Zhang YQ, Bailey AM, Matthies HJG (2001). Drosophila fragile X-related gene regulates the MAP1B homolog Futsch to control synaptic structure and function. *Cell*.

[26] Tessier CR, Broadie K (2008). Drosophila fragile X mental retardation protein developmentally regulates activity-dependent axon pruning. *Development*.

[27] Antar LN, Bassell GJ (2003). Sunrise at the synapse: the FMRP mRNP shaping the synaptic interface. *Neuron*.

[28] Auerbach BD, Bear MF (2010). Loss of the fragile X mental retardation protein decouples metabotropic glutamate receptor dependent priming of long-term potentiation from protein synthesis. *Journal of Neurophysiology*.

[29] Costa-Mattioli M, Sossin WS, Klann E, Sonenberg N (2009). Translational control of long-lasting synaptic plasticity and memory. *Neuron*.

[30] Huber KM, Gallagher SM, Warren ST, Bear MF (2002). Altered synaptic plasticity in a mouse model of fragile X mental retardation. *Proceedings of the National Academy of Sciences of the United States of America*.

[31] Pan L, Woodruff E, Liang P, Broadie K (2008). Mechanistic relationships between *Drosophila* fragile X mental retardation protein and metabotropic glutamate receptor A signaling. *Molecular and Cellular Neuroscience*.

[32] Waung MW, Huber KM (2009). Protein translation in synaptic plasticity: mGluR-LTD, fragile X. *Current Opinion in Neurobiology*.

[33] Zhang YQ, Broadie K (2005). Fathoming fragile X in fruit flies. *Trends in Genetics*.

[34] Antar LN, Afroz R, Dictenberg JB, Carroll RC, Bassell GJ (2004). Metabotropic glutamate receptor activation regulates fragile x mental retardation protein and FMR1 mRNA localization differentially in dendrites and at synapses. *Journal of Neuroscience*.

[35] Bear MF (2005). Therapeutic implications of the mGluR theory of fragile X mental retardation. *Genes, Brain and Behavior*.

[36] Bear MF, Dölen G, Osterweil E, Nagarajan N (2008). fragile X: translation in action. *Neuropsychopharmacology*.

[37] Bear MF, Huber KM, Warren ST (2004). The mGluR theory of fragile X mental retardation. *Trends in Neurosciences*.

[38] Dölen G, Bear MF (2008). Role for metabotropic glutamate receptor 5 (mGluR5) in the pathogenesis of fragile X syndrome. *Journal of Physiology*.

[39] Dölen G, Carpenter RL, Ocain TD, Bear MF (2010). Mechanism-based approaches to treating fragile X. *Pharmacology and Therapeutics*.

[40] Dölen G, Osterweil E, Rao BSS (2007). Correction of fragile X syndrome in mice. *Neuron*.

[41] Meredith RM, de Jong R, Mansvelder HD (2011). Functional rescue of excitatory synaptic transmission in the developing hippocampus in Fmr1-KO mouse. *Neurobiology of Disease*.

[42] Pan L, Zhang YQ, Woodruff E, Broadie K (2004). The Drosophila fragile X gene negatively regulates neuronal elaboration and synaptic differentiation. *Current Biology*.

[43] Repicky S, Broadie K (2009). Metabotropic glutamate receptor-mediated use-dependent down-regulation of synaptic excitability involves the fragile X mental retardation protein. *Journal of Neurophysiology*.

[44] Bolduc FV, Bell K, Cox H, Broadie KS, Tully T (2008). Excess protein synthesis in *Drosophila* fragile X mutants impairs long-term memory. *Nature Neuroscience*.

[45] Choi CH, McBride SMJ, Schoenfeld BP (2010). Age-dependent cognitive impairment in a *Drosophila* fragile X model and its pharmacological rescue. *Biogerontology*.

[46] McBride SMJ, Choi CH, Wang Y (2005). Pharmacological rescue of synaptic plasticity, courtship behavior, and mushroom body defects in a *Drosophila* model of fragile X syndrome. *Neuron*.

[47] Pan L, Broadie KS (2007). *Drosophila* fragile X mental retardation protein and metabotropic glutamate receptor a convergently regulate the synaptic ratio of ionotropic glutamate receptor subclasses. *Journal of Neuroscience*.

[48] Levenga J, de Vrij FMS, Oostra BA, Willemsen R (2010). Potential therapeutic interventions for fragile X syndrome. *Trends in Molecular Medicine*.

[49] Wang LW, Berry-Kravis E, Hagerman RJ (2010). fragile X: leading the way for targeted treatments in autism. *Neurotherapeutics*.

[50] Berry-Kravis E, Sumis A, Hervey C (2008). Open-label treatment trial of lithium to target the underlying defect in fragile X syndrome. *Journal of Developmental and Behavioral Pediatrics*.

[51] Bilousova TV, Dansie L, Ngo M (2009). Minocycline promotes dendritic spine maturation and improves behavioural performance in the fragile X mouse model. *Journal of Medical Genetics*.

[52] Paribello C, Tao L, Folino A (2010). Open-label add-on treatment trial of minocycline in fragile X syndrome. *BMC Neurology*.

[53] Siller SS, Broadie K (2011). Neural circuit architecture defects in a *Drosophila* model of fragile X syndrome are alleviated by minocycline treatment and genetic removal of matrix metalloproteinase. *Disease Models and Mechanisms*.

[54] Utari A, Chonchaiya W, Rivera SM (2010). Side effects of minocycline treatment in patients with fragile X syndrome and exploration of outcome measures. *American journal on intellectual and developmental disabilities*.

[55] Kim HS, Suh YH (2009). Minocycline and neurodegenerative diseases. *Behavioural Brain Research*.

[56] Griffin MO, Fricovsky E, Ceballos G, Villarreal F (2010). Tetracyclines: a pleitropic family of compounds with promising therapeutic properties. Review of the literature. *American Journal of Physiology - Cell Physiology*.

[57] Agrawal SM, Lau L, Yong VW (2008). MMPs in the central nervous system: where the good guys go bad. *Seminars in Cell and Developmental Biology*.

[58] Ethell IM, Ethell DW (2007). Matrix metalloproteinases in brain development and remodeling: synaptic functions and targets. *Journal of Neuroscience Research*.

[59] Page-McCaw A, Ewald AJ, Werb Z (2007). Matrix metalloproteinases and the regulation of tissue remodelling. *Nature Reviews Molecular Cell Biology*.

[60] Page-McCaw A, Serano J, Santë JM, Rubin GM (2003). *Drosophila* matrix metalloproteinases are required for tissue remodeling, but not embryonic development. *Developmental Cell*.

[61] Brundula V, Rewcastle NB, Metz LM, Bernard CC, Yong VW (2002). Targeting leukocyte MMPs and transmigration minocycline as a potential therapy for multiple sclerosis. *Brain*.

[62] van den Bosch L, Tilkin P, Lemmens G, Robberecht W (2002). Minocycline delays disease onset and mortality in a transgenic model of ALS. *NeuroReport*.

[63] Zhu S, Stavrovskaya IG, Drozda M (2002). Minocycline inhibits cytochrome c release and delays progression of amyotrophic lateral sclerosis in mice. *Nature*.

[64] Wang X, Zhu S, Drozda M (2003). Minocycline inhibits caspase-independent and -dependent mitochondrial cell death pathways in models of Huntington’s disease. *Proceedings of the National Academy of Sciences of the United States of America*.

[65] Wu DC, Jackson-Lewis V, Vila M (2002). Blockade of microglial activation is neuroprotective in the 1-methyl-4-phenyl-1,2,3,6-tetrahydropyridine mouse model of Parkinson disease. *Journal of Neuroscience*.

[66] Choi Y, Kim HS, Shin KY (2007). Minocycline attenuates neuronal cell death and improves cognitive impairment in Alzheimer’s disease models. *Neuropsychopharmacology*.

[67] Rivera S, Khrestchatisky M, Kaczmarek L, Rosenberg GA, Jaworski DM (2010). Metzincin proteases and their inhibitors: foes or friends in nervous system physiology?. *Journal of Neuroscience*.

[68] Crocker SJ, Pagenstecher A, Campbell IL (2004). The TIMPs tango with MMPs and more in the central nervous system. *Journal of Neuroscience Research*.

[69] Stetler-Stevenson WG (2008). Tissue inhibitors of metalloproteinases in cell signaling: metalloproteinase-independent biological activities. *Science signaling*.

[70] Rotschafer SE, Trujillo MS, Dansie LE, Ethell IM, Razak KA (2012). Minocycline treatment reverses ultrasonic vocalization production deficit in a mouse model of fragile X syndrome. *Brain Research*.

[71] Bakker CE, Verheij C, Willemsen R (1994). Fmr1 knockout mice: a model to study fragile X mental retardation. *Cell*.

[72] Kooy RF, D’Hooge R, Reyniers E (1996). Transgenic mouse model for the fragile X syndrome. *American Journal of Medical Genetics*.

[73] Heulens I, Kooy F (2011). fragile X syndrome: from gene discovery to therapy. *Frontiers in Bioscience*.

[74] Hinton VJ, Brown WT, Wisniewski K, Rudelli RD (1991). Analysis of neocortex in three males with the fragile X syndrome. *American Journal of Medical Genetics*.

[75] Rudelli RD, Brown WT, Wisniewski K (1985). Adult fragile X syndrome. Clinico-neuropathologic findings. *Acta Neuropathologica*.

[76] Ethell IM, Pasquale EB (2005). Molecular mechanisms of dendritic spine development and remodeling. *Progress in Neurobiology*.

[77] Knott GW, Holtmaat A, Wilbrecht L, Welker E, Svoboda K (2006). Spine growth precedes synapse formation in the adult neocortex *in vivo*. *Nature Neuroscience*.

[78] Marrs GS, Green SH, Dailey ME (2001). Rapid formation and remodeling of postsynaptic densities in developing dendrites. *Nature Neuroscience*.

[79] Ziv NE, Smith SJ (1996). Evidence for a role of dendritic filopodia in synaptogenesis and spine formation. *Neuron*.

[80] Comery TA, Harris JB, Willems PJ (1997). Abnormal dendritic spines in fragile X knockout mice: maturation and pruning deficits. *Proceedings of the National Academy of Sciences of the United States of America*.

[81] Nimchinsky EA, Oberlander AM, Svoboda K (2001). Abnormal development of dendritic spines in FMR1 knock-out mice. *Journal of Neuroscience*.

[82] Bilousova TV, Rusakov DA, Ethell DW, Ethell IM (2006). Matrix metalloproteinase-7 disrupts dendritic spines in hippocampal neurons through NMDA receptor activation. *Journal of Neurochemistry*.

[83] Bushey D, Tononi G, Cirelli C (2009). The drosophila fragile X mental retardation gene regulates sleep need. *Journal of Neuroscience*.

[84] Coffee RL, Tessier CR, Woodruff EA, Broadie K (2010). fragile X mental retardation protein has a unique, evolutionarily conserved neuronal function not shared with FXR1P or FXR2P. *DMM Disease Models and Mechanisms*.

[85] Dockendorff TC, Su HS, McBride SMJ (2002). Drosophila lacking dfmr1 activity show defects in circadian output and fail to maintain courtship interest. *Neuron*.

[86] Gatto CL, Broadie K (2008). Temporal requirements of the fragile X mental retardation protein in the regulation of synaptic structure. *Development*.

[87] Gatto CL, Broadie K (2009). Temporal requirements of the fragile X mental retardation protein in modulating circadian clock circuit synaptic architecture. *Front Neural Circuits*.

[88] Gatto CL, Broadie K (2009). The fragile X mental retardation protein in circadian rhythmicity and memory consolidation. *Molecular Neurobiology*.

[89] Inoue SB, Shimoda M, Nishinokubi I (2002). A role for the *Drosophila* fragile X-related gene in circadian output. *Current Biology*.

[90] Morales J, Hiesinger PR, Schroeder AJ (2002). Drosophila fragile X protein, DFXR, regulates neuronal morphology and function in the brain. *Neuron*.

[91] Tessier CR, Broadie K (2011). The fragile X mental retardation protein developmentally regulates the strength and fidelity of calcium signaling in *Drosophilas* mushroom body neurons. *Neurobiology of Disease*.

[92] Zhang YQ, Matthies HJG, Mancuso J (2004). The *Drosophila* fragile X-related gene regulates axoneme differentiation during spermatogenesis. *Developmental Biology*.

[93] Callan MA, Cabernard C, Heck J, Luois S, Doe CQ, Zarnescu DC (2010). fragile X protein controls neural stem cell proliferation in the *Drosophila* brain. *Human molecular genetics*.

[94] Cziko AMJ, McCann CT, Howlett IC (2009). Genetic modifiers of dFMR1 encode RNA granule components in *Drosophila*. *Genetics*.

[95] Epstein AM, Bauer CR, Ho A, Bosco G, Zarnescu DC (2009). *Drosophila* fragile X protein controls cellular proliferation by regulating cbl levels in the ovary. *Developmental Biology*.

[96] Estes PS, O’Shea M, Clasen S, Zarnescu DC (2008). fragile X protein controls the efficacy of mRNA transport in *Drosophila* neurons. *Molecular and Cellular Neuroscience*.

[97] Chang S, Bray SM, Li Z (2008). Identification of small molecules rescuing fragile X syndrome phenotypes in *Drosophila*. *Nature Chemical Biology*.

[98] Zarnescu DC, Shan G, Warren ST, Jin P (2005). Come FLY with us: toward understanding fragile X syndrome. *Genes, Brain and Behavior*.

[99] Coffee RL, Williamson AJ, Adkins CM, Gray MC, Page TL, Broadie K *In vivo* neuronal function of the fragile X mental retardation protein is regulated by phosphorylation.

[100] Michaluk P, Wawrzyniak M, Alot P (2011). Influence of matrix metalloproteinase MMP-9 on dendritic spine morphology. *Journal of Cell Science*.

[101] Page-McCaw A (2008). Remodeling the model organism: matrix metalloproteinase functions in invertebrates. *Seminars in Cell and Developmental Biology*.

[102] Glasheen BM, Kabra AT, Page-McCaw A (2009). Distinct functions for the catalytic and hemopexin domains of a *Drosophila* matrix metalloproteinase. *Proceedings of the National Academy of Sciences of the United States of America*.

[103] Glasheen BM, Robbins RM, Piette C, Beitel GJ, Page-McCaw A (2010). A matrix metalloproteinase mediates airway remodeling in *Drosophila*. *Developmental Biology*.

[104] Michaluk P, Kaczmarek L (2007). Matrix metalloproteinase-9 in glutamate-dependent adult brain function and dysfunction. *Cell Death and Differentiation*.

[105] Nagy V, Bozdagi O, Matynia A (2006). Matrix metalloproteinase-9 is required for hippocampal late-phase long-term potentiation and memory. *Journal of Neuroscience*.

[106] Tian L, Stefanidakis M, Ning L (2007). Activation of NMDA receptors promotes dendritic spine development through MMP-mediated ICAM-5 cleavage. *Journal of Cell Biology*.

[107] Gawlak M, Górkiewicz T, Gorlewicz A, Konopacki FA, Kaczmarek L, Wilczynski GM (2009). High resolution in situ zymography reveals matrix metalloproteinase activity at glutamatergic synapses. *Neuroscience*.

[108] Konopacki FA, Rylski M, Wilczek E (2007). Synaptic localization of seizure-induced matrix metalloproteinase-9 mRNA. *Neuroscience*.

[109] Duchossoy Y, Horvat JC, Stettler O (2001). MMP-related gelatinase activity is strongly induced in scar tissue of injured adult spinal cord and forms pathways for ingrowing neurites. *Molecular and Cellular Neuroscience*.

[110] Moore CS, Crocker SJ (2012). An alternate perspective on the roles of TIMPs and MMPs in pathology. *The American Journal of Pathology*.

[111] Le AP, Friedman WJ (2012). Matrix metalloproteinase-7 regulates cleavage of pro-nerve growth factor and is neuroprotective following kainic acid-induced seizures. *The Journal of Neuroscience*.

[112] de Maria A, Solaro C, Abbruzzese M, Primavera A (1997). Minocycline for symptomatic neurosyphilis in patients allergic to penicillin. *The New England Journal of Medicine*.

[113] Hayashi N, Kawashima M (2011). Efficacy of oral antibiotics on acne vulgaris and their effects on quality of life: a multicenter randomized controlled trial using minocycline, roxithromycin and faropenem. *Journal of Dermatology*.

[114] Moult PR, Corrêa SAL, Collingridge GL, Fitzjohn SM, Bashir ZI (2008). Co-activation of p38 mitogen-activated protein kinase and protein tyrosine phosphatase underlies metabotropic glutamate receptor-dependent long-term depression. *Journal of Physiology*.

[115] Pi R, Li W, Lee NTK (2004). Minocycline prevents glutamate-induced apoptosis of cerebellar granule neurons by differential regulation of p38 and Akt pathways. *Journal of Neurochemistry*.

